# Role of aquaporin-4 polarization in extracellular solute clearance

**DOI:** 10.1186/s12987-024-00527-7

**Published:** 2024-03-26

**Authors:** Laura Bojarskaite, Sahar Nafari, Anne Katrine Ravnanger, Mina Martine Frey, Nadia Skauli, Knut Sindre Åbjørsbråten, Lena Catherine Roth, Mahmood Amiry-Moghaddam, Erlend A. Nagelhus, Ole Petter Ottersen, Inger Lise Bogen, Anna E. Thoren, Rune Enger

**Affiliations:** 1 GliaLab and Letten Centre, Division of Anatomy, Department of Molecular Medicine, Institute of Basic Medical Sciences, University of Oslo, P.O.B. 1103, Oslo, 0317 Norway; 2 Department of Neurology, Oslo University Hospital, Oslo, 0027 Norway; 3 Division of Anatomy, Department of Molecular Medicine, Institute of Basic Medical Sciences, University of Oslo, Oslo, 0317 Norway; 4 Section for Drug Abuse Research, Department of Forensic Sciences, Oslo University Hospital, P.O. Box N-4950, Nydalen, Oslo, 0424 Norway

**Keywords:** AQP4, Glymphatic, Waste clearance, Astrocyte, Glia, Syntrophin, Dystrophin

## Abstract

Waste from the brain has been shown to be cleared via the perivascular spaces through the so-called glymphatic system. According to this model the cerebrospinal fluid (CSF) enters the brain in perivascular spaces of arteries, crosses the astrocyte endfoot layer, flows through the parenchyma collecting waste that is subsequently drained along veins. Glymphatic clearance is dependent on astrocytic aquaporin-4 (AQP4) water channels that are highly enriched in the endfeet. Even though the polarized expression of AQP4 in endfeet is thought to be of crucial importance for glymphatic CSF influx, its role in extracellular solute clearance has only been evaluated using non-quantitative fluorescence measurements. Here we have quantitatively evaluated clearance of intrastriatally infused small and large radioactively labeled solutes in mice lacking AQP4 (*Aqp4*^*–/–*^) or lacking the endfoot pool of AQP4 (*Snta1*^*–/–*^). We confirm that *Aqp4*^*–/–*^ mice show reduced clearance of both small and large extracellular solutes. Moreover, we find that the *Snta1*^*–/–*^ mice have reduced clearance only for the 500 kDa [^3^H]dextran, but not 0.18 kDa [^3^H]mannitol suggesting that polarization of AQP4 to the endfeet is primarily important for clearance of large, but not small molecules. Lastly, we observed that clearance of 500 kDa [^3^H]dextran increased with age in adult mice. Based on our quantitative measurements, we confirm that presence of AQP4 is important for clearance of extracellular solutes, while the perivascular AQP4 localization seems to have a greater impact on clearance of large versus small molecules.

**Supplementary Information:**

The online version contains supplementary material available at 10.1186/s12987-024-00527-7.

## Main points

Solute clearance is reduced in mice lacking AQP4

Polarization of AQP4 to the endfeet may have a greater impact on clearance of large versus small molecules

Clearance of large but not small solutes is correlated with age within adult age

## Introduction

A growing body of evidence suggests that excess interstitial fluid (ISF), solutes and waste products are cleared from the brain parenchyma via perivascular spaces through the so-called glymphatic system [1, 2]. The original groundwork of brain waste clearance pathways was performed by Cserr and colleagues in 1970–1980s who observed that waste from the brain was cleared in a size-independent manner along perivascular spaces [3–5]. The glymphatic model extends these observations and describes that flow of extracellular fluid in the brain involves three components: (1) cerebrospinal fluid (CSF) influx into the brain along the perivascular spaces (PVS) of penetrating arterioles, (2) fluid flow through the parenchyma, and (3) efflux of interstitial fluid and waste products from the brain in the PVS along veins. The initial glymphatic study and several follow up studies have shown that extracellular solute clearance is facilitated by the astrocytic water channels aquaporin-4 (AQP4), that are highly enriched in astrocytic endfeet [2, 6]. Later studies have demonstrated that glymphatic flow is driven by arterial blood vessel dynamics [7–9], is more effective in sleep [10–13] and under certain types of anesthesia [14], and is less effective in advanced age [15].

It is unclear through which mechanism the passive water channel AQP4 affects glymphatic flow. A role for AQP4 in brain fluid flow dynamics was already first suggested with its discovery in astrocytes in the late 1990s [16], and the direct role for AQP4 in clearance of injected fluid was first suggested in 2004 by Papadopolous et al. [17]. In the context of the glymphatic system, it has been suggested that the pronounced polarization of AQP4 to the perivascular endfeet in particular is important for both tracer influx [6] and efflux [18], but this has only been evaluated with non-quantitative fluorescence microscopy techniques. Also, the overall importance of AQP4 in brain waste clearance has been questioned by others in the field [19]. Hence, there is a need for quantitative assessment of the role of AQP4 polarization on solute clearance from the brain.

Here we aimed to quantitatively evaluate the role of AQP4 and AQP4 polarization to the astrocytic endfeet on glymphatic efflux of extracellular solutes. We measured clearance of 0.18 kDa [^3^H]mannitol and 500 kDa [^3^H]dextran infused through an acutely implanted cannula into the striatum of mice devoid of AQP4 (*Aqp4*^*–/–*^ mice) and mice that lack the AQP4 anchoring protein α-syntrophin, which results in 90% loss of the perivascular pool of AQP4 (*Snta1*^*–/–*^ mice) [20–22]. Although acute intrastriatal cannula implantation has been shown to impair glymphatic flow [23], we aimed to use similar techniques to what was used in the original glymphatic system reports [2, 10] to directly compare how solute clearance was affected by overall AQP4 removal and AQP4 polarization to the endfeet. We found that removal of AQP4 attenuated clearance of both 0.18 kDa [^3^H]mannitol and 500 kDa [^3^H]dextran, similar to the original reports [2]. Surprisingly, clearance of large and small solutes differed in *Snta1*^*–/–*^ mice. We found that the clearance of large 500 kDa [^3^H]dextran, but not the small 0.18 kDa [^3^H]mannitol was reduced in *Snta1*^*–/–*^ mice. Lastly, we evaluated how age within normal adult range affected the clearance of large and small molecules. We found that the clearance of 500 kDa [^3^H]dextran increased with age in adult mice, while clearance of 0.18 kDa [^3^H]mannitol remained unaffected. Our quantitative measures confirm that AQP4 is important for clearance of extracellular solutes and add novel insights that the polarization of AQP4 to the astrocytic endfeet and age within the adult range are more important for the clearance of large than small solutes.

## Results

### Deletion of AQP4 reduces extracellular solute clearance

**Fig. 1:**
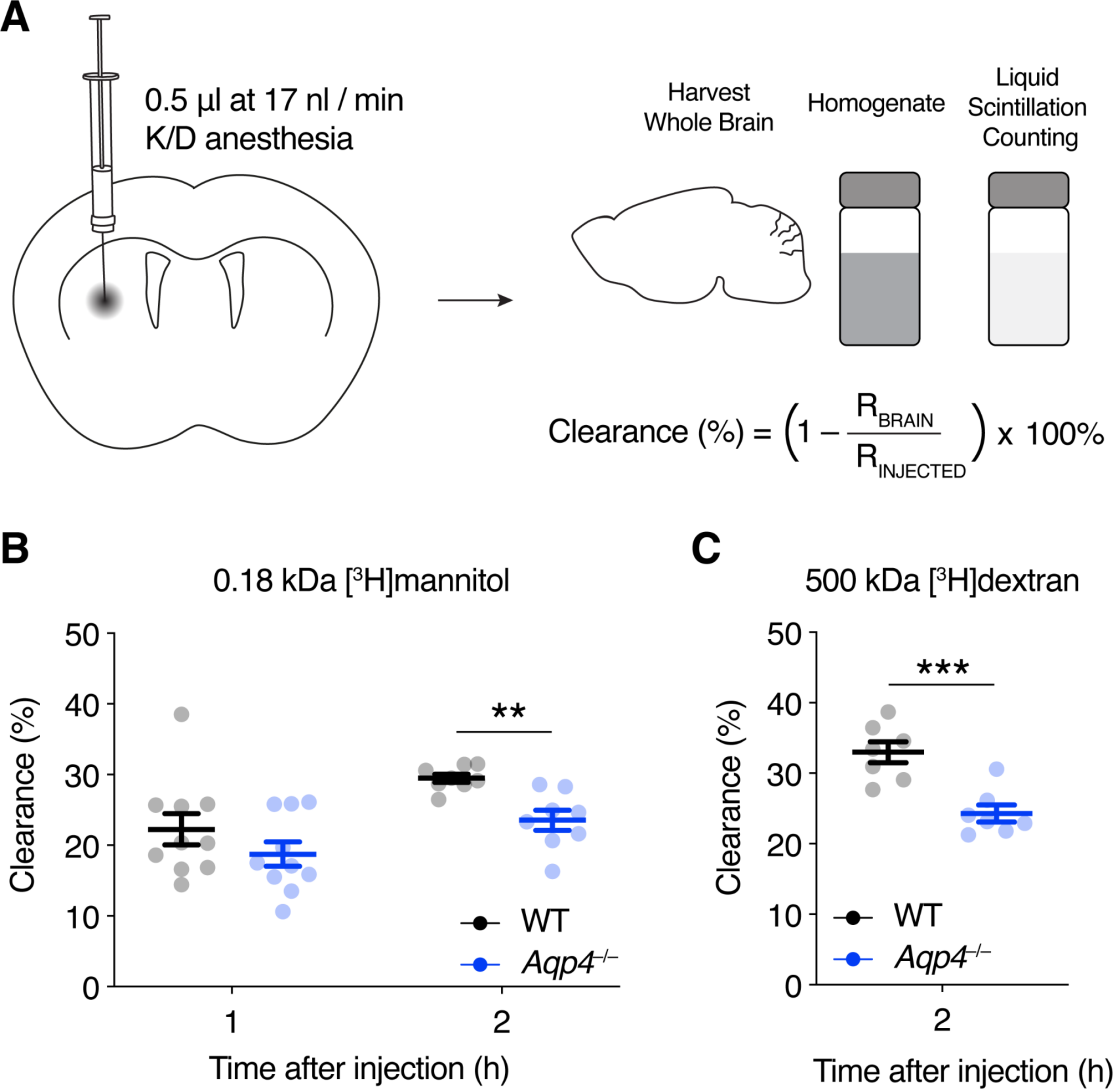
Clearance of extracellular solutes is reduced in *Aqp4*^*–/–*^ mice. (**A**) Schematic diagram illustrating the quantification of extracellular solute clearance from the brain parenchyma. Radioactive tracers (0.18 kDa [^3^H]mannitol and 500 kDa [^3^H]dextran) infused into the striatum of anesthetized mice. Whole brains were harvested, homogenized and radioactivity counted by liquid scintillation counting. The percentage clearance was calculated based on total injected radioactivity (R_INJECTED_) and the remaining radioactivity in the brain (R_BRAIN_). (**B**) Clearance of intrastriatal 0.18 kDa [^3^H]mannitol in age-matched *Aqp4*^*–/–*^ and WT mice 1 and 2 h after infusion. *n* = 10 at 1 h; *n* = 8 at 2 h per genotype. Average ± SD age: for 1 h – *Aqp4*^*–/–*^ 105 ± 12 days, WT 83 ± 12 days; for 2 h – for *Aqp4*^*–/–*^ 99 ± 14, for WT 91 ± 15. (C) Clearance of intrastriatal 500 kDa [^3^H]dextran in age-matched *Aqp4*^*–/–*^ and WT mice 2 h after infusion. *n* = 7 per genotype. Average ± SD age: *Aqp4*^*–/–*^ 105 ± 27 days, WT 104 ± 27 days. Data represented as mean ± s.e.m, ** = *p* < 0.01, *** = *p* < 0.001, mixed effects linear regression model (see Materials and Methods)

To evaluate the dependence of intraparenchymal solute clearance on the astrocytic water channel AQP4, we infused the radiolabeled tracers 0.18 kDa [^3^H]mannitol or 500 kDa [^3^H]dextran into the striatum of anesthetized age-matched *Aqp4*^*–/–*^ and WT mice and assessed the overall brain content of radioactivity left at 1 or 2 h after infusion (Fig. 1a). We defined percentage clearance as 1 minus radioactivity left in the brain divided by radioactivity injected, similar to the original reports. Mice were kept under anesthesia for the entire duration of the experiment. A volume of 0.5 µl and slow infusion speed of 17 nl/min was used to minimize hydrostatic pressure gradients and increases in intracranial pressure, which could affect fluid and solute flow in the parenchyma [24, 25]. In *Aqp4*^*–/–*^ mice, the clearance of 0.18 kDa [^3^H]mannitol and 500 kDa [^3^H]dextran 2 h after infusion was reduced compared to WT mice (Fig. 1b–c, *p* = 0.11 for 1 h [^3^H]mannitol, *p* = 0.0022 for 2 h [^3^H]mannitol, *p* = 0.00071 for 2 h 500 kDa [^3^H]dextran), in line with the original publication by Iliff et al. [2]. However, compared to Iliff et al., we observed a more moderate effect of AQP4 removal on clearance for 0.18 kDa [^3^H]mannitol (27% vs. 70%) after 2 h, and did not detect any difference in clearance between the genotypes 1 h after infusion.

### Perivascular AQP4 may have a greater impact on clearance of large versus small molecules

**Fig. 2:**
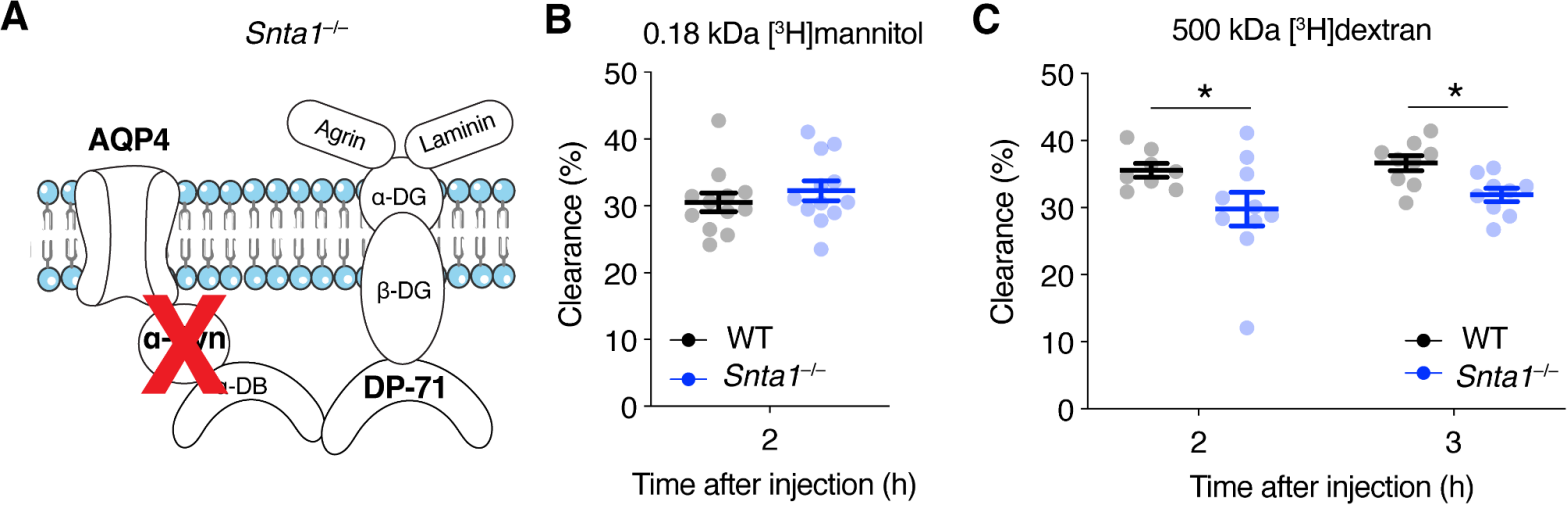
The role of perivascular AQP4 in the clearance of extracellular solutes. (**A**) *Snta1*^*–/–*^ mice have severely attenuated level of AQP4 in endfeet due to the lack of α-syntrophin, which is an integral part of the anchoring of AQP4. (**B**) Clearance of intrastriatal [^3^H]mannitol in age-matched *Snta1*^*–/–*^ and WT mice 2 h after infusion. Average ± SD age: *Snta1*^*–/–*^ 95 ± 13 days, WT 93 ± 14 days. *n* = 12 mice per genotype. (**C**) Clearance of intrastriatal 500 kDa [^3^H]dextran in age-matched *Snta1*^*–/–*^ and WT mice 2 and 3 h after infusion. *n* = 8 for WT and *n* = 10 for *Snta1*^*–/–*^ at 2 h, *n* = 9 mice per genotype at 3 h. Average ± SD age: for 2 h – *Snta1*^*–/–*^ 120 ± 12 days, WT 124 ± 17 days; for 3 h – for *Snta1*^*–/–*^ 113 ± 22, for WT 122 ± 21. Data represented as mean ± s.e.m, * = *p* < 0.05, ** = *p* < 0.01, mixed effects linear regression model (see Materials and Methods)

To quantitatively evaluate the role of the highly polarized subcellular distribution pattern of astrocytic AQP4 in extracellular waste removal, we evaluated clearance of radioactively labeled 0.18 kDa [^3^H]mannitol and 500 kDa [^3^H]dextran tracers in *Snta1*^*–/–*^ mice that exhibits a strong reduction in perivascular AQP4. *Snta1*^–/–^ mice lack the AQP4 anchoring molecule α-syntrophin and have a ~90% reduction in perivascular AQP4, but no change in total AQP4 expression levels [21, 22, 26] (Fig. 2a). The clearance of 0.18 kDa [^3^H]mannitol was not significantly different between *Snta1*^–/–^mice and WT controls 2 h after infusion (Fig. 2b, *p* = 0.35). Interestingly, *Snta1*^–/–^ mice showed a reduced clearance of 500 kDa [^3^H]dextran compared to WT controls 2 h (*p* = 0.013) and 3 h (*p* = 0.039) after infusion (Fig. 2c). In conclusion, the perivascular endfoot pool of AQP4 seems to play a role in the clearance of large, but not small molecules.

### Clearance of extracellular solutes is size-independent

**Fig. 3:**
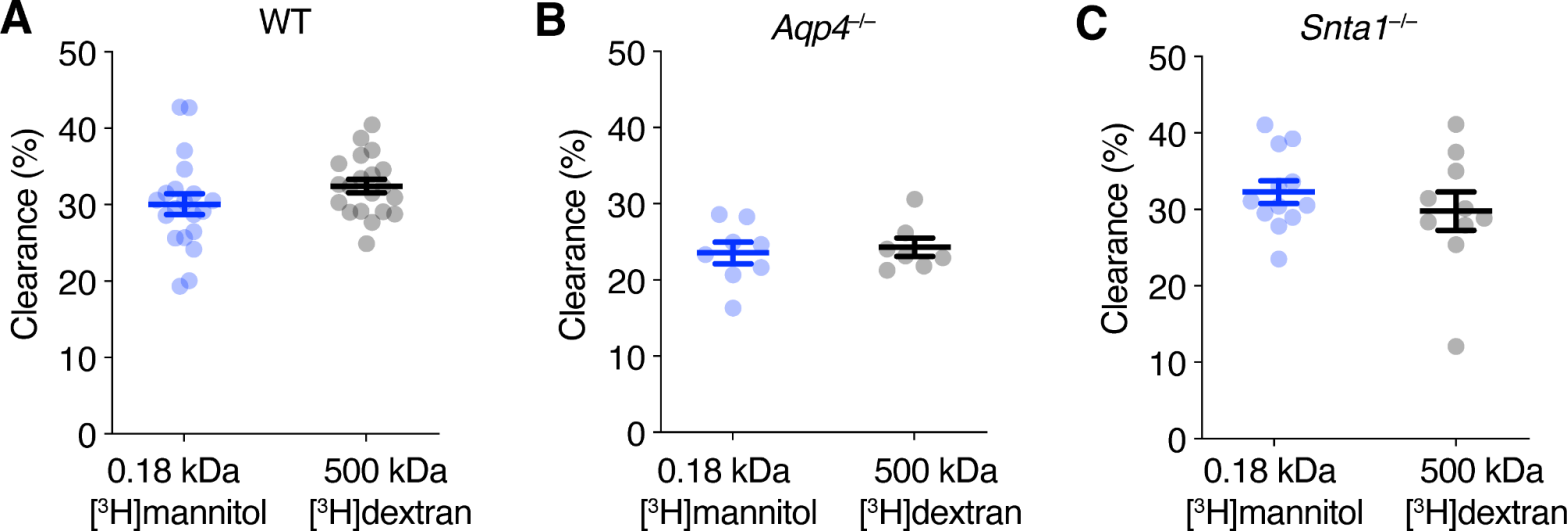
Clearance of extracellular solutes is size independent. Clearance of intrastriatal 0.18 kDa [^3^H]mannitol and 500 kDa [^3^H]dextran 2 h after infusion in (**A**) WT mice, average ± SD age: [^3^H]mannitol 95 ± 13 days, [^3^H]dextran 93 ± 14 days. (**B**) *Aqp4*^*–/–*^ mice that lack AQP4, average ± SD age: [^3^H]mannitol 95 ± 13 days, [^3^H]dextran 93 ± 14 days. and (**C**) *Snta1*^–/–^ mice which have have severely attenuated level of AQP4 in endfeet due to the lack of α-syntrophin, average ± SD age: [^3^H]mannitol 95 ± 13 days, [^3^H]dextran 93 ± 14 days. Data represented as mean ± s.e.m. WT: *n* = 20 mice per group; *Aqp4*^*–/–*^ mice: *n* = 8 for [^3^H]mannitol, *n* = 7 for [^3^H]dextran; *Snta1*^–/–^ mice: *n* = 12 for [^3^H]mannitol, *n* = 10 for [^3^H]dextran; unpaired two tailed *t*-test

One debated aspect of the glymphatic model concerns the mechanism of the parenchymal solute transport. In the original glymphatic publication, the authors propose convective bulk flow, as clearance did not depend on solute size [2]. This was in accordance with the pioneering reports on solute movement in the brain showing that clearance was independent of size [3]. Others argue that convective flow in the perivascular spaces suffice to explain the size independence of tracer clearance, and that in the parenchyma, diffusion over short distances towards the nearest blood vessels is the predominant mechanism of transport [25, 27–31]. In line with previous findings, we find that the clearance of 0.18 kDa [^3^H]mannitol and 500 kDa [^3^H]dextran was not significantly different (Fig. 3a) 2 h after infusion despite the large differences in the molecular sizes and hydrodynamic radii of the molecules (0.18 kDa and 0.4 nm for [^3^H]mannitol; 500 kDa and 16 nm for [^3^H]dextran). Such size-independence of extracellular solute clearance was observed not only in WT mice, but also in *Aqp4*^*–/–*^ and *Snta1*^*–/–*^ mice (Fig. 3b,c).

### Increased clearance of large, but not small extracellular molecules is correlated with older age in adult mice

**Fig. 4:**
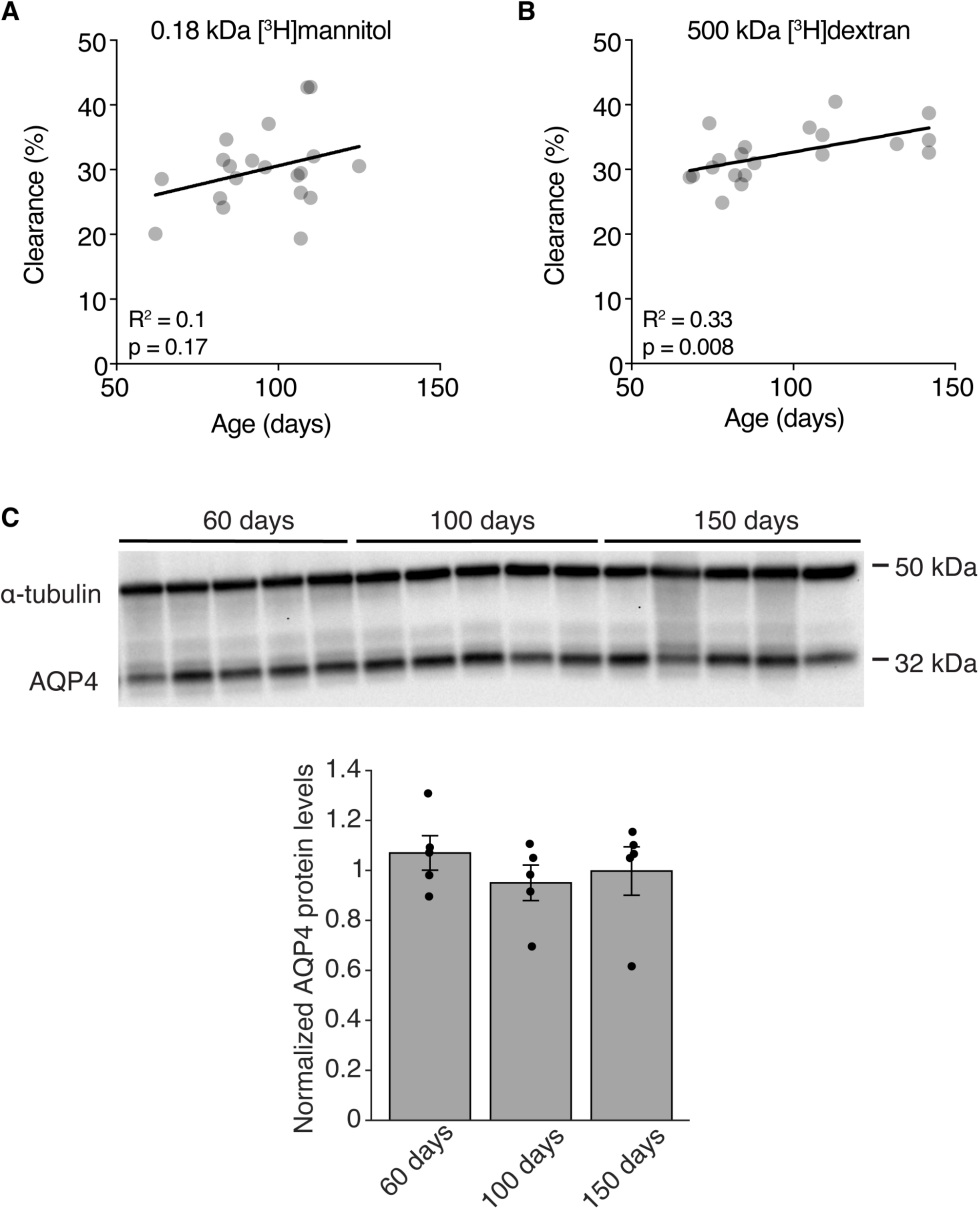
The effect of age on extracellular solute clearance. (**A–B**) Correlation between age and clearance of 0.18 kDa [^3^H]mannitol (*n* = 20) or 500 kDa [^3^H]dextran (*n* = 20) in WT mice (All WT controls from Figs. 1 and 2 pooled). (**C**) Representative immunoblots and quantification expressed in densitometric values of total protein lysates from striatum of 60, 100 and 150 day old WT mice. *n* = 5 for each group. (**A–B)** analyzed by linear regression

Glymphatic influx depends on many physiological variables such as age, heart rate, body position and respiration rate [14, 15, 32]. We measured how efflux is affected by animal age within the range of normal adult mice 60–150 days in WT mice. We found that the age of the mouse within adult range did not affect clearance of [^3^H]mannitol (Fig. 4a) while the clearance of [^3^H]dextran increases with age (Fig. 4b). To investigate whether this change in clearance was due to age-dependent differences in expression of AQP4, we performed quantitative western blot analyses. No age-difference was found in levels of AQP4 across the different age groups (Fig. 4c). With the present data we cannot rule out differences in AQP4 polarization with increasing age, but given the modest effect of completely abolishing the polarization of AQP4 in *Snta1*^*–/–*^ mice we do not expect that a potential change in polarization with age could explain the increase in clearance with age that we observe. Taken together, these data suggest that small and large molecules might be cleared by different mechanisms where removal of large molecules could depend on age.

## Discussion

Loss of AQP4 polarization is a hallmark of a wide range of brain pathologies, such as epilepsy [33], stroke [34], idiopathic normal pressure hydrocephalus [35] and Alzheimer’s disease [36]. Therefore, it is crucial to understand whether loss of endfoot AQP4 affects the clearance of extracellular solutes. Using a quantitative method of radioactive tracer infusion to the striatum of anesthetized mice, we here show that the subcellular polarization of AQP4 to the astrocytic endfeet may have a greater impact on clearance of large versus small molecules. Moreover, in accordance with the literature [2–4], we found a similar clearance for 0.18 kDa [^3^H]mannitol with a hydrodynamic radius of 0.4 nm and the ~2800 times larger 500 kDa [^3^H]dextran with a hydrodynamic radius of 16 nm. Lastly, we show that clearance of large molecules increased with age within the adult age range, while clearance of small molecules was not affected.

Astrocytic endfeet almost completely ensheath the blood vessels of the brain and likely represent a barrier between the PVS and the parenchyma hindering free flow of fluids and solutes [37]. Iliff et al. [2] suggested that the highly concentrated expression of AQP4 in the endfeet was important for the glymphatic system, and recent semi-quantitative studies of the distribution of injected fluorescent tracers in *Snta1*^*–/–*^ mice have suggested both an attenuated influx [6] and efflux [18] of tracers in these mice. Partly in agreement with these reports, using quantitative assessment of clearance of radioactive tracers, we demonstrate that perivascular pool of AQP4 is more important for glymphatic clearance of large than small extracellular solutes. We found no difference in the clearance of [^3^H]mannitol between *Snta1*^*–/–*^ and WT mice, while for 500 kDa [^3^H]dextran we found a reduced clearance in the *Snta1*^*–/–*^ mice 2 and 3 h after injection. This suggests that the highly polarized expression of AQP4 cannot account for all the effects of AQP4 for brain solute clearance. We did not observe a significant increase in clearance between 2 and 3 h timepoints. The fact that tracer clearance is faster initially, is in line with existing literature [2, 10, 23], and could be explained by a model where tracer clearance is concentration dependent, suggesting a role for diffusion. However, similar clearance rates between vastly different molecular sizes of tracers observed by us and shown in a range of previous studies [2, 3], suggest that convection plays a role. Likely our observations are best explained by a combination of parenchymal diffusion, which could be one of the mechanisms contributing to faster initial clearance, and perivascular convection, accounting for size-independence of overall clearance [27]. Lastly, we cannot rule out potential issues with variability or technical concerns such as infusion triggered local pressure increases or clumping of the 500 kDa dextran molecules which could clog the drainage pathways.

It is unclear how AQP4 contributes to glymphatic clearance. It is tempting to think that the channels facilitate transcellular fluid flow across the endfoot sleeve based on the dense clustering of these channels at the endfoot membrane facing the vessel, but it is unclear how such transcellular fluid flow could occur and what would be the driving forces acting upon this system. The resistance to flow has been modeled to be considerably lower between endfeet than through AQP4 [27]. If fluid flow in between adjacent endfeet is the main exit route for fluid from the PVS to the parenchyma and vice versa, one could imagine that these slots are subject to gating mechanisms that are affected by the presence of AQP4. Yet, to the best of our knowledge, no study has been able to address whether endfoot coverage of vessels in vivo can vary or is subjected to regulation. It has also been suggested that the increased size of the extracellular space in *Aqp4*^*–/–*^ mice could play a role [19]. However, in this case one would rather expect an increased clearance in the *Aqp4*^–/–^ mice, given the proposed role of increased extracellular space during sleep in the glymphatic system [10]. Similarly, in *Snta1*^*–/–*^ mice, a higher baseline extracellular space has been shown, albeit to a lesser extent than in *Aqp4*^*–/–*^ mice [38]. However, solute influx from the CSF to the interstitial space seems to be AQP4 independent as shown by Smith et al., via application of fluorescent dextrans to the brain surface under constant pressure [39]. The concentration of AQP4 in endfeet has been reported to be higher around capillaries and veins than around arterioles [2]. This observation might suggest that AQP4 is more important for processes not linked with hydrostatic forces associated with perivascular bulk flow around arterioles. One avenue worth exploring in future studies may be to what extent the capillaries contribute to ISF production in the brain, as already suggested in the pioneering work of Cserr et al. [40]. Even though equally as elusive as the role of AQP4 in the hydrostatic framework of the glymphatic system, potentially AQP4 could play a role in facilitating BBB production of ISF. Finally, a large and growing body of literature suggests that endfeet possess the machinery to modulate vessel tone, even though to what extent endfeet partake in vascular control remains controversial [41–43]. In Haidey et al. they propose that stretch-sensitive TRPV4 channels in the astrocytic endfeet play a role in modulating vasomotion [43]. Potentially AQP4 may play a role in the same signaling pathways, enabling the endfeet to react to vascular dynamics, which again may influence the driving forces of the glymphatic system. Future studies should address the mechanistic underpinnings of how AQP4 affects clearance of solutes, for example evaluate whether AQP4 could regulate the morphology of endfeet, the inter-endfoot gaps or vascular dynamics; and selectively manipulate AQP4 expression and polarization levels in arterioles, venules and capillaries and evaluate how this contributes to clearance of small and large molecules.

The role of AQP4 in interstitial waste clearance as originally proposed by Iliff et al., and reported in numerous follow-up studies was challenged by Smith et al. who did not detect any differences between *Aqp4*^–/–^ and WT mice [19]. Our findings are in line with the original Iliff et al. study, as we find that extracellular solute removal is reduced in mice lacking AQP4. However, compared with Iliff et al., we find a somewhat smaller effect of AQP4 on clearance. The reason for these discrepancies with the original study is not entirely clear, but could be due to differences in volume of tracer injected (0.5 µl in our study vs. 1 µl in Iliff et al.), different injection methods, or different protocols for radioactivity counting.

Intraparenchymal injection pressures could explain different conclusions about glymphatic flow in different studies [6]. Arguably, injection pressures could easily exceed any potential hydrostatic driving force created by arterial pulsations, confounding assessment of direction of flow in the parenchyma, and potentially also exit pathways. Hence, we did go to great lengths in order to minimize any pressure artifacts by using a very low speed of infusion and a minimal injected volume (0.5 µl over 30 min). Moreover, glymphatic flow is reduced after acute cannulation [23]. While we cannot exclude that tissue responses to acute cannulation, such as BBB disruption, inflammation, swelling, global CNS responses such as intracranial pressure or recovery of lost CSF volume, or steric effects might differ between genotypes and could contribute to different clearance results, we aimed to use similar methods as in the original glymphatic system report that described the effect of AQP4 [2] for direct comparison of the effect of AQP4 polarization to the endfeet. Future studies should confirm our findings using less invasive methods such as the real-time in vivo clearance assay for quantification of glymphatic efflux in which tracers are infused via a chronic implanted cannula and clearance is measured in periphery avoiding post mortem artifacts [23].

One surprising finding from our dataset was the significant positive correlation between age and clearance. Previous studies have demonstrated that clearance is negatively affected by age [15]. However, these studies are not directly comparable to ours as they investigated young (2–3 months) middle aged (10–12 months) and old mice (18 months), whereas we analyzed the dependency on age in mice within our experimental groups (ranging from 60 to 150 days of age). From our data, it is apparent that one should take the age of mice carefully into consideration, even within groups of adult mice, as this may influence the outcome. One potential mechanism through which clearance could depend on age is expression of AQP4. Even though we did not observe any overall differences in the levels of AQP4 between the groups of 60, 100 and 150 days old mice, it could be that polarization of AQP4 to the endfeet or that selective AQP4 expression around arterioles, venules and capillaries varies in this age range.

To conclude, using a quantitative method we confirm findings from previous semi-quantitative fluorescence studies that both the presence and subcellular distribution of AQP4 is important for brain solute clearance, and add that large and small solutes might be cleared by partly different mechanisms, where removal of large molecules partly depends on AQP4 polarization to the endfeet and increases with age within adult range.

## Materials and methods

### Mice

Adult male WT mice (C57BL/6J, Janvier Labs), *Aqp4*^−/−^ mice [20] and *Snta1*^−/−^ mice [26], of 8 weeks to 6 months of age were used. The transgenic mice were back-crossed into a C57BL/6J background for at least 10 generations. All mice, except WT mice were bred at the University of Oslo. Mice were housed on a 12 h light:12 h dark cycle (lights on at 8 AM), 1–4 mice per cage. Experiments were carried out in accordance with the guidelines published in the European Communities Council Directive of 24 November 1986 (86/609/EEC). All procedures were approved by the Animal Use and Care Committee of the Institute of Basic Medical Sciences, the Faculty of Medicine at the University of Oslo and the Norwegian Food Safety Authority (project number: 11942).

### Tracers

Radioactively labeled [^3^H]mannitol (ARC-ART Mannitol, D-(2-3H), 1 µCi/µl) and 500 kDa [^3^H]dextran (ARC-ART 1373 − 250, 1 µCi/µl) (Larodan AB) were dissolved at a ratio of 1:10 in isosmotic artificial CSF, containing (in mM): 124 NaCl, 2 KCl, 1.25 KH_2_PO_4_, 2 MgSO_4_, 2 CaCl_2_, 26 NaHCO_3_, and 12 glucose, pH 7.3).

### Intrastriatal tracer infusion

Mice were anesthetized intraperitoneally with a mixture of medetomidine (0.3 mg/kg) and ketamine (40 mg/kg), placed in a stereotactic frame on a heating pad at 37 °C and supplied with O_2_ to maintain pO_2_ > 85%. pO_2_ and heart rate were continuously monitored by a PhysioSuite apparatus (Kent Scientific). If pO_2_ values at any time dropped below 85%, mice were excluded from the study. An incision was made to expose the skull and a small burr hole was drilled at coordinates of 0.5 mm posterior and 2.3 mm lateral relative to bregma. A Hamilton syringe (Neuros syringe, 32GA, 0.5 µl) was loaded with content to be injected, inserted to a depth of 3 mm from the surface of the brain and left for 30 min before infusion to let the needle tract seal. The tracer of interest was infused at a rate of 17 nl/min up to a total volume of 0.5 µl using an 11 Elite Nanomite pump (Harvard Apparatus). 1, 2 or 3 h after infusion of radioactive tracers, the mice were decapitated and brains stored at -20 °C. The needle was left in place for the entire duration of the experiment to prevent tracer loss via needle tract.

### Radioactivity quantification

The whole brains were thawed and homogenized in 2.5 mL 50 mM Tris HCl buffer (pH 7.4) with 0.5% Triton, using a glass Teflon homogenizer (15 strokes, 900 rpm; Eurostar Power-B, IKA-Werke). Duplicates of 100 and 250 µl were transferred to glass scintillation vials (Perkin Elmer, 20 mL) and 15 mL Hionic-Fluor (Perkin Elmer) was added. The samples were shaken for 60 s and stored in darkness for 30 min before radioactivity was counted in a liquid scintillation analyzer (Tri-Carb 2810TR; PerkinElmer). Brain radioactivity was expressed as the percentage of the total injected radioactivity left in the brain.

### Western blot

Brains of 60 days old (*n* = 5), 100 days old (*n* = 5) and 150 days old (*n* = 5) C57BL/6 mice were dissected, and striata homogenized in RIPA buffer with freshly added 1X protease (Sigma-Aldrich; Cat# S8820) and phosphatase inhibitors (Roche Life Sciences, Cat# 4906845001) and incubated on ice for 30 min with occasional vortexing before centrifugation at 14,000 rpm at 4 °C for 15 min. The supernatant was collected as total protein, and concentrations measured using a Pierce™ BCA protein assay (Thermo Fisher, Waltham, MA, USA). Protein samples were heated in 1× Laemmli sample buffer at 37 °C for 10 min and separated on 4–20% Criterion™ 18-well TGX gels (BioRad; Cat# 5671094) using the Criterion™ (BioRad) Tris-glycine system at 185 V for 1 h 15 min at 4 °C. Proteins were transferred to 0.2 μm Immun-Blot PVDF membranes by wet blotting at 100 V for 30 min at 4 °C (BioRad). Uniform transfer of proteins was verified by reversible Ponceau S staining (0.1%w/v, 1% acetic acid, Sigma-Aldrich; Cat# P7170). 10 µg of protein was used for detection of AQP4. Membranes were blocked for 1 h at RT (using 5% BSA diluted in 1× Tris-buffered saline) before overnight incubation at 4 °C with primary antibody against AQP4 (Rabbit anti-AQP4, 1:2000, Sigma-Aldrich, A5971). On the following day, secondary antibody conjugated to HRP was applied for 1 h at RT (Donkey anti-rabbit HRP, 1:25000, Amersham, GE Life Sciences) and the blot was washed and developed. Protein expression of α-tubulin (Rabbit anti-a-tubulin, 1:5000, Abcam, ab4074) was used for normalization. Immunoreactive bands were detected by SuperSignal™ West Pico Chemiluminescent Substrate (Thermo Fisher Scientific; Cat#: 34580) on the ChemiDoc™ Touch Imaging System (BioRad) and bands quantified as arbitrary background-subtracted density units in Image Studio Lite (Ver 5.2, Licor Biosciences, Nebraska). Normalization was performed by dividing intensities of protein bands of interest with the control band intensity for their respective lane. The obtained values were transferred to SPSS Version 26 (SPSS, Chicago, IL) and compared using independent samples *t*-test. Data are presented as mean ± SD and *p < 0.05 was considered as significant.

### Statistical analyses

Clearance of radioactively labeled tracer was estimated with a linear mixed effects regression model where clearance left in the brain (expressed as a fraction of the injected value) was considered the response value, while genotype and timepoint and their interaction were considered fixed effects, and finally date of experiment was included as a mixed effects term. Since we found an effect of age on clearance when evaluating all the data points sampled from WT mice, we also ran the statistical models adding age as a covariate. This did not change which comparisons were significant, most likely because the groups were closely age-matched. Goodness of fit was assessed by symmetrical distribution of residuals. To assess the robustness of the findings the results were cross-validated by randomly excluding ~1/3 of the samples in each group and re-running the model (see Supplementary Fig. 1 for histograms of estimates per genotype/time point if this reduced model is run 300 times). For comparisons only between two groups, Kolmogorov-Smirnov and Shapiro-Wilk tests were used to confirm that the data were normally distributed, and hence comparisons between different size tracers (Fig. 3) were performed by unpaired two-tailed t-tests. Supplementary Table 1 includes the radioactivity counting values for the injected and the remaining radioactivity, age and date of experiment for each animal in each experimental group.

### Electronic supplementary material

Below is the link to the electronic supplementary material.

Supplementary Material 1

Supplementary Material 2

## Data Availability

The data that support the findings of this study are available from the corresponding author upon reasonable request.
