# Supplementary material for: Role of aquaporin-4 polarization in extracellular solute clearance
Source: Fluids Barriers CNS. 2024 Mar 26;21:28. doi: 10.1186/s12987-024-00527-7 (PMC10964559; doi:10.1186/s12987-024-00527-7)
Supplement: Supplementary file 1 — Supplementary Material 1 [file 12987_2024_527_MOESM1_ESM.pdf]

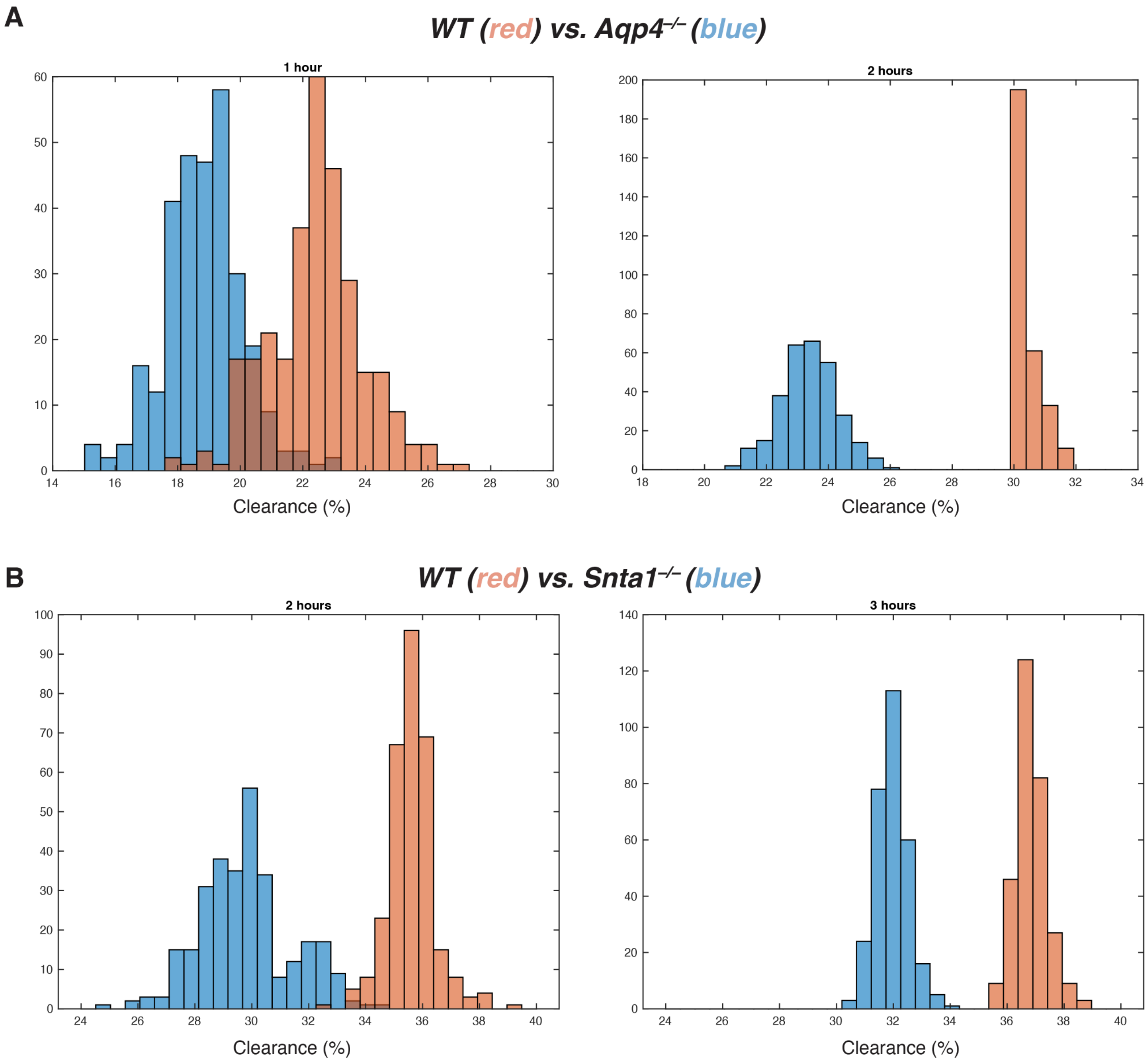

Supplementary Figure 1. Cross-validation of statistical modeling. (A) The statistical model underlying Figure 1B is run while randomly omitting ~30% of datapoints in each experimental group, for 300 iterations. The histograms show model estimates for genotype and timepoints. (B) Same for the dataset in Figure 2C.
